# Supplementary material for: Continuous antiretroviral therapy induces progressive senescence-like reprogramming of alveolar macrophages
Source: Front Immunol. 2026 Apr 22;17:1805936. doi: 10.3389/fimmu.2026.1805936 (PMC13144088; doi:10.3389/fimmu.2026.1805936)
Supplement: Supplementary file 1 [file DataSheet1.pdf]

Supplementary Figures

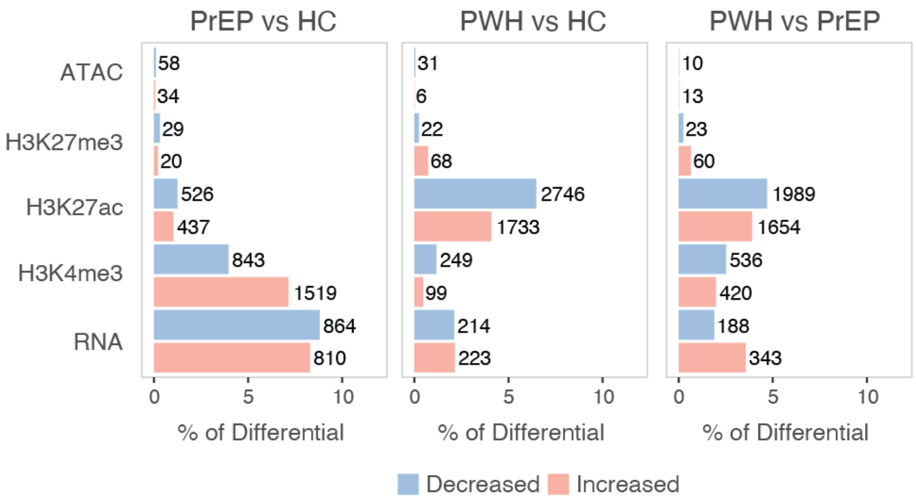

Fig. S1. Differential epigenetic and transcriptomic features between groups.

Differential epigenetic and transcriptomic features between groups are shown for the three baseline comparisons. The bar plots show the proportion of differential regions/genes at an FDR of 10% and absolute log2FC > 0.2 relative to the total number of tested regions/genes for time on ART in PrEP vs HC (left), PWH vs HC (center) and PWH vs PrEP (right). The absolute number of differential regions/genes are shown next to the bars. Decreased and increased DNA accessibility, histone marks, and gene expression are shown in blue and red, respectively.

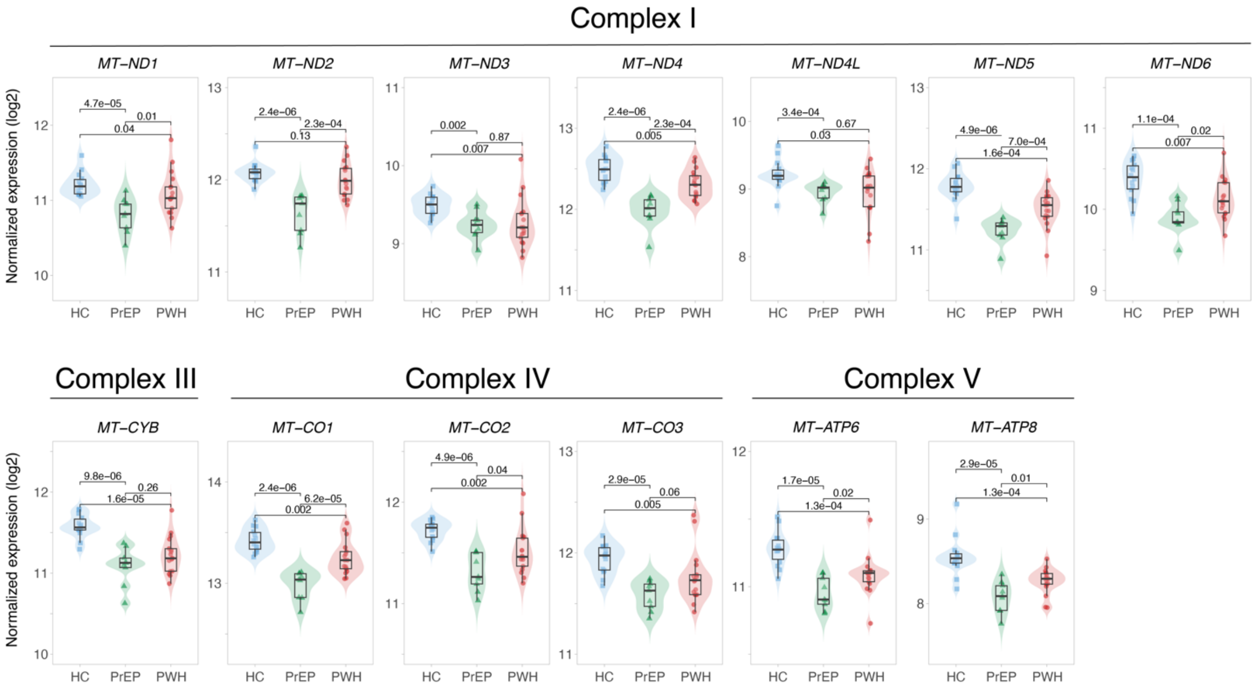

Fig. S2. Reduced expression of mitochondrial genes encoding enzymes of the electron transport chain.

Normalized gene expression per subject and group for mitochondrial genes encoding proteins of the human electron transport chain. *p*-values for the Wilcoxon test comparing differences in median expressions between groups are shown at the top.

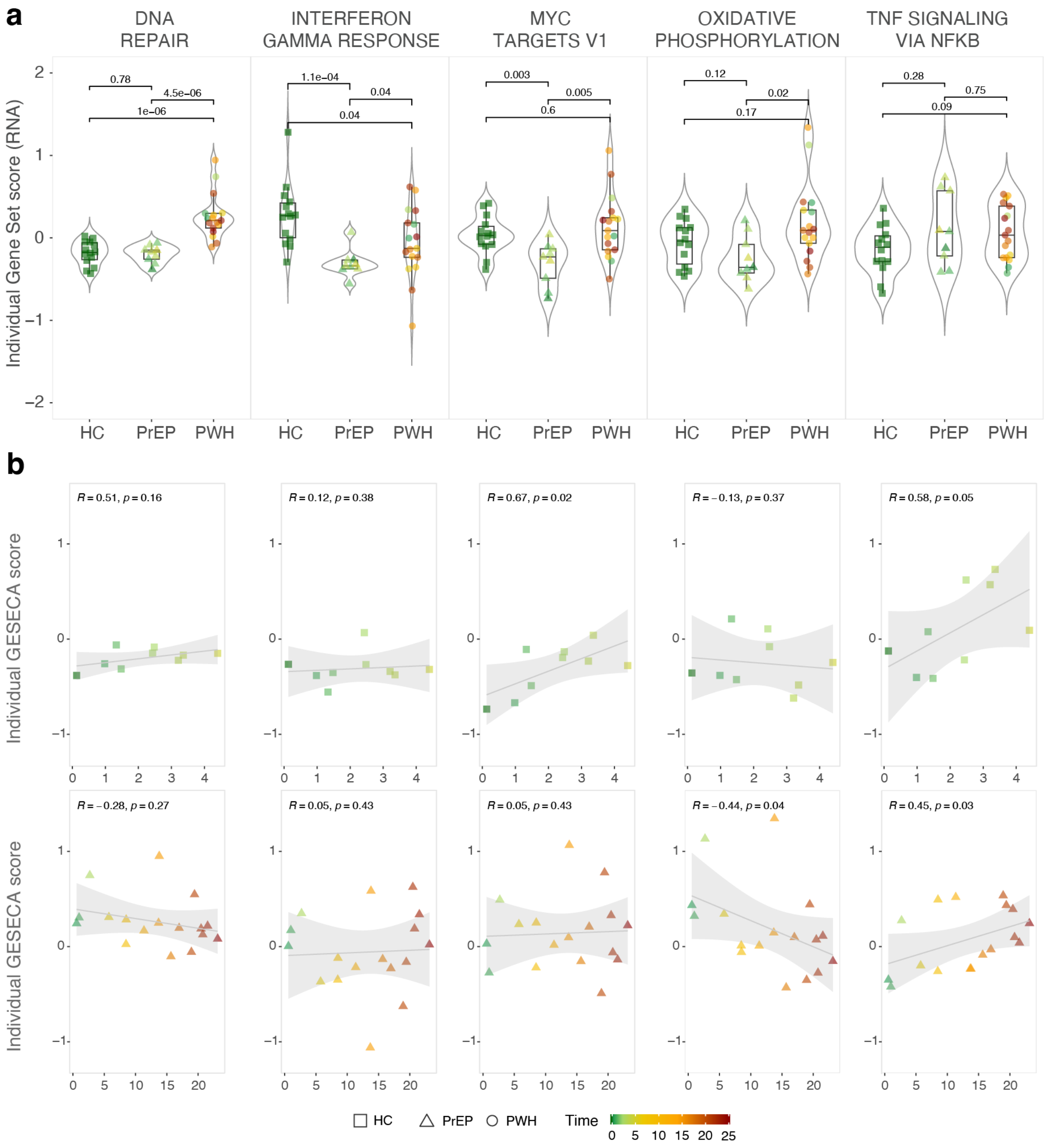

Fig. S3. Comparison between GESECA scores and phenotypic groups as in Fig. 2C indicating time on ART.

(a) GESECA scores for the transcriptomic assay for each subject are plotted on the y-axis separated by phenotypic group on the x-axis. The *p*-values shown at the top are for the group compassions shown in Fig. 2C. The colour shades indicate the length of time on ART for PrEP and for PWH. **(b)** Correlation plots between GESECA scores and time on ART for the groups PrEP and PWH

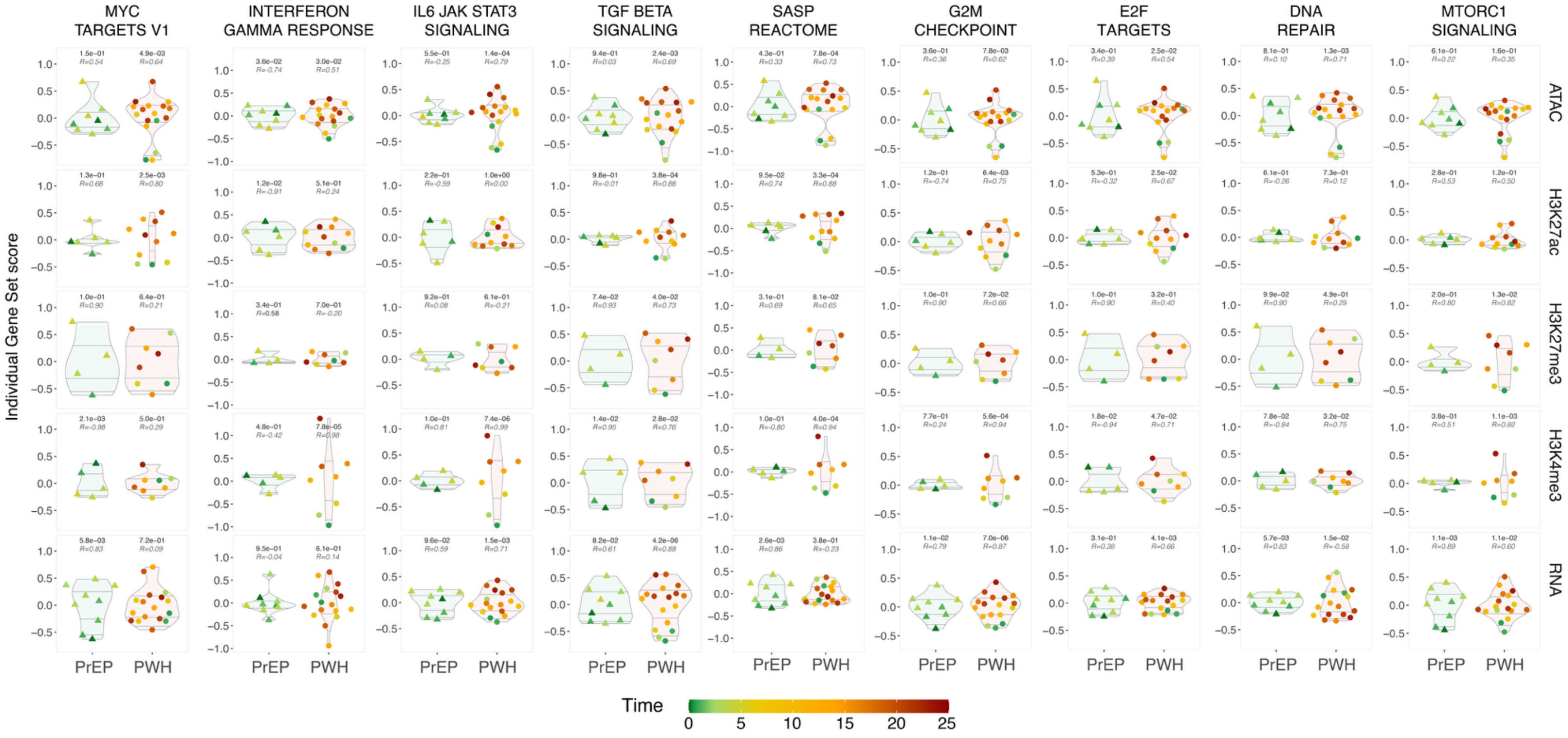

Fig. S4. **Per subject GESECA score for eight pathways affected by time as a complement to Fig. 4B.**

Each dot represents an individual with colour shading indicating time on ART for PrEP or for PWH. GESECA scores per individual were plotted on the y-axis and subjects were separated by groups on the x-axis. The quantiles are shown as violins for reference of the distribution. Pearson correlation coefficients and corresponding *p*-values are shown for the comparisons between time on ART in PrEP and PWH with the individual GESECA scores for all tested assays.

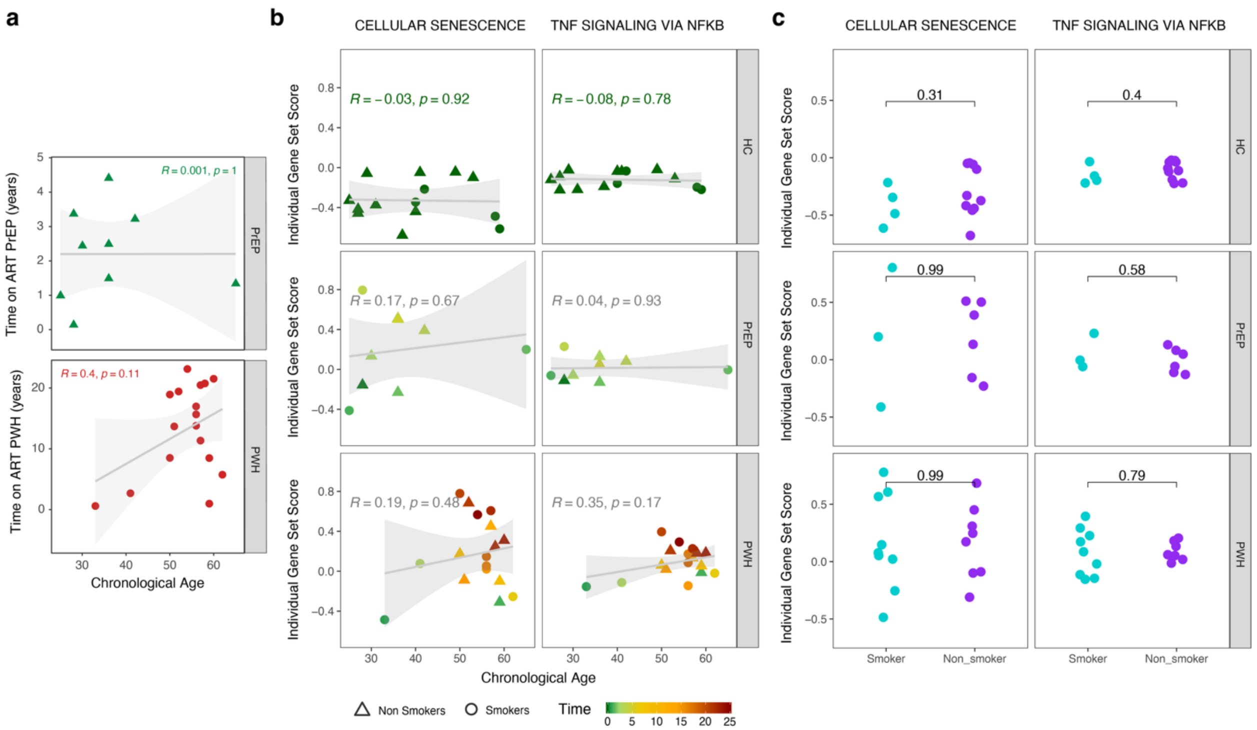

Fig. S5. Effect of chronological age and smoking status on ART associated pathways.

(**a**) Correlation plot between chronological age and time on ART for people on PrEP (top) or for PWH (bottom). (**b**) Correlation plot between individual gene set scores of the RNA assay for two main pathways associated with time and chronological age. (**c**) Comparison of individual gene set scores of the RNA assay and smoking status for two main pathways associated with time on ART.

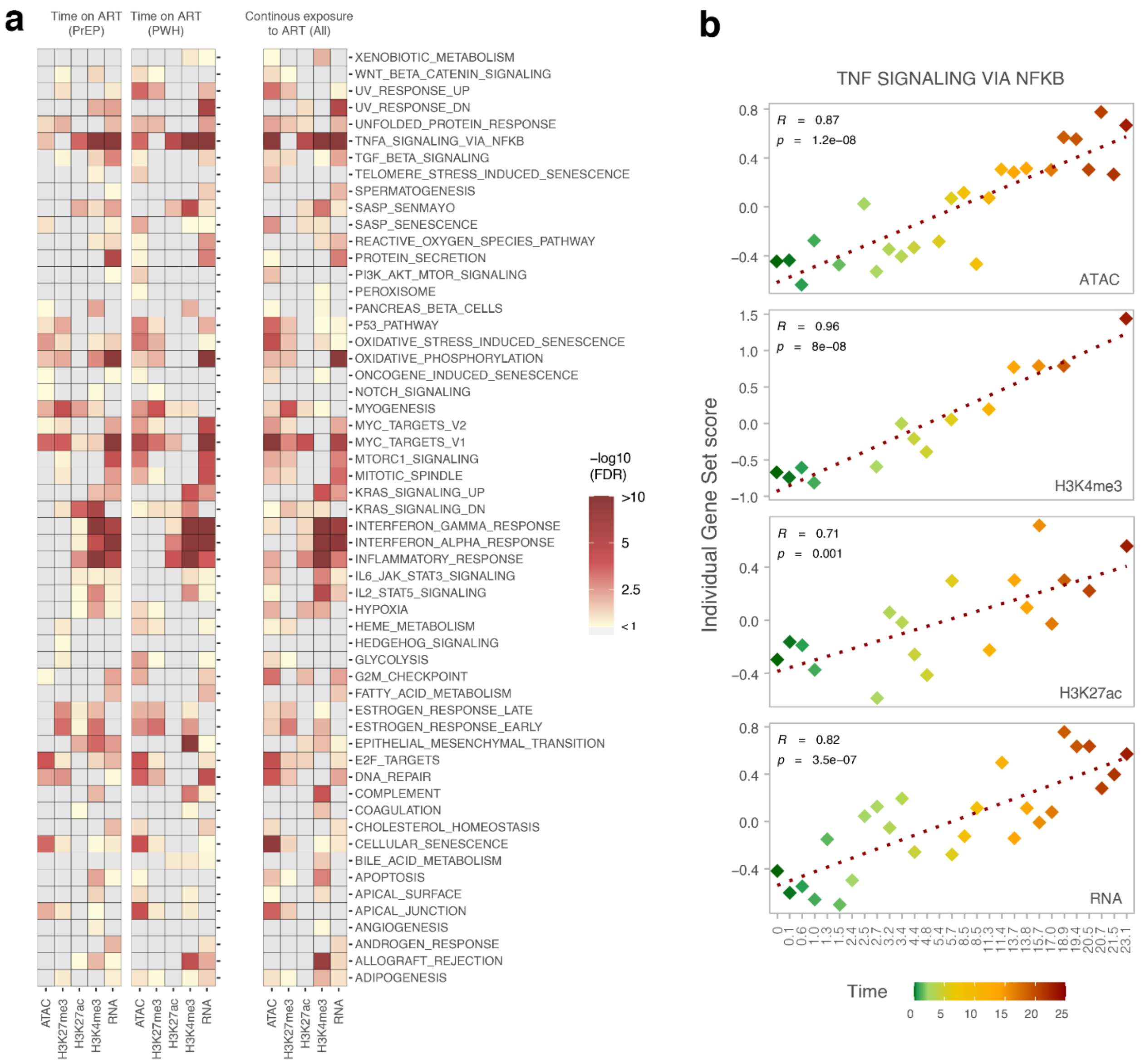

Fig. S6. GESECA association with continuous exposure to ART**.**

(**a**) GESECA for Hallmark pathways for time on ART in PrEP (left), in PWH (center), and continuous exposure to ART irrespective of the group (right). FDR for all tested gene sets is shown as a heatmap with increased significance from yellow to red.(**b**) Correlation of individual gene set scores (y-axis) for the TNF signaling pathway with length of ART exposure (x-axis). Pearson correlation R and corresponding *p*-values are shown at the top left of the plots.

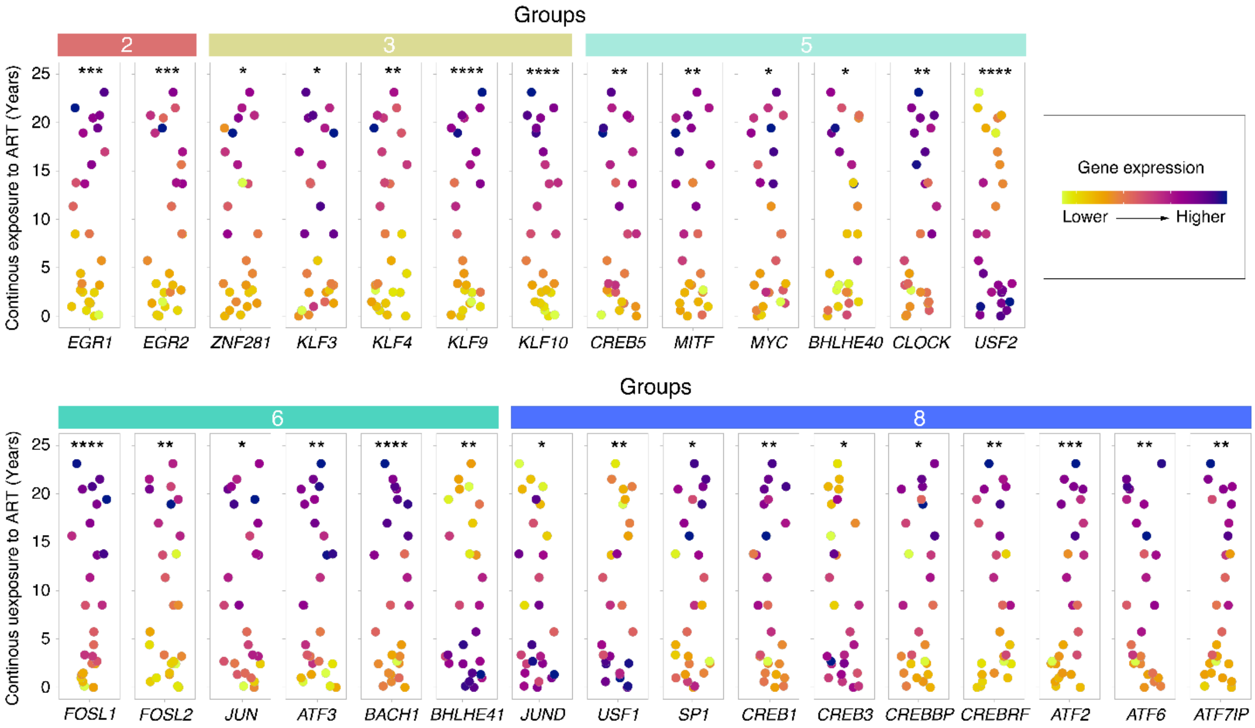

Fig. S7. Coordinated increased gene expression in AM of transcription factors with motifs associated with continuous exposure to ART.

Boxplots showing the time of continuous exposure to ART on the y-axes for genes encoding transcription factors (TF) shown in Fig. 4d. Each dot indicates the continuous exposure to ART for all subjects. The boxes at the top of the plots show the corresponding groups for the TF gene. FDR significance for the continuous exposure to ART. *, FDR < 10%, ** FDR < 1%, *** FDR < 0.1% and, **** FDR < 0.01%.

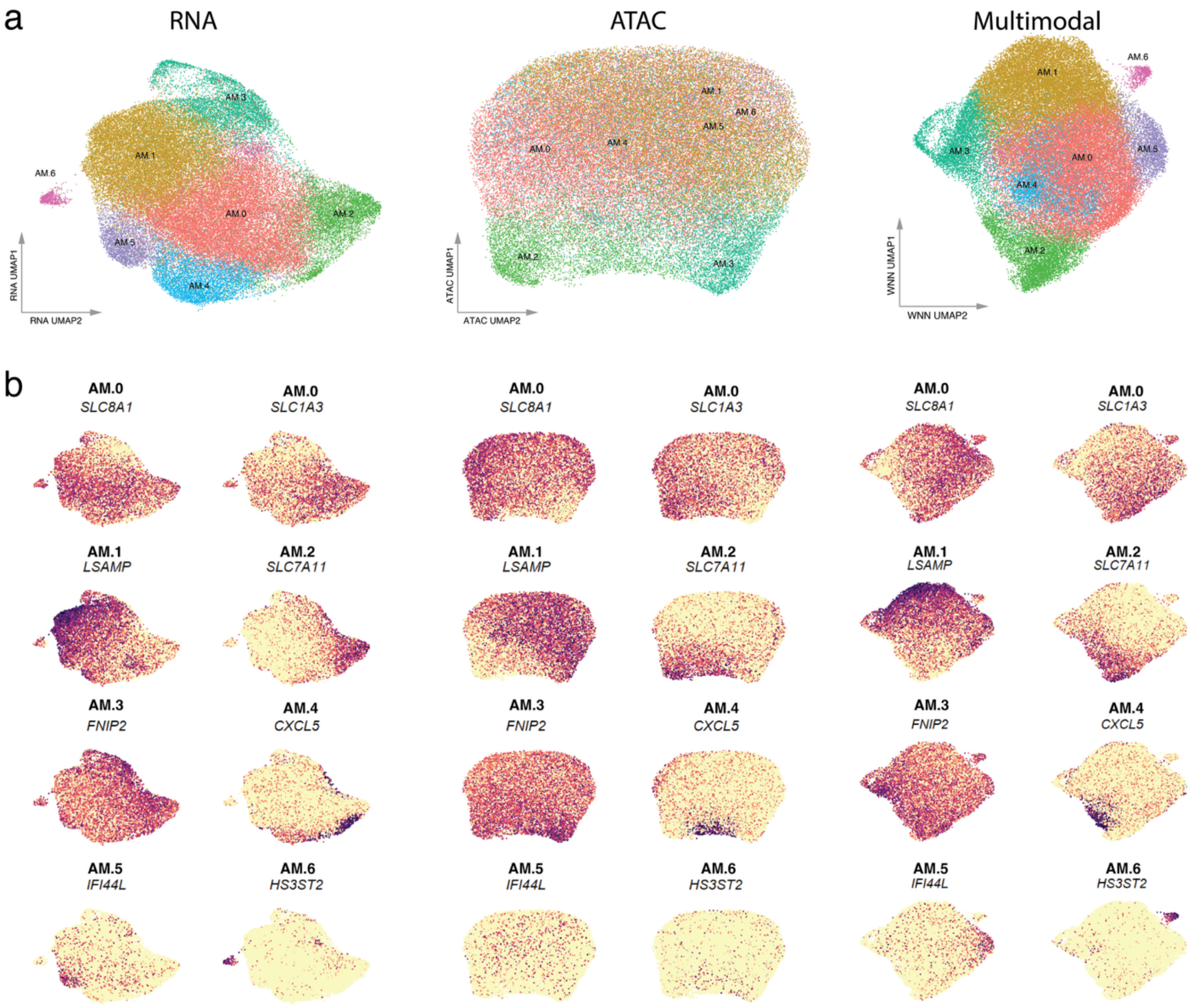

Fig. S8. Annotation of AM subpopulation with single nucleus multiomics.

(a) UMAPs for each modality (RNA and ATAC), as well as the multimodal weighted nearest neighbor (WNN) integration, showing the annotated AM subpopulations defined via single nucleus multimodality. (b) UMAPs displaying the expression of top cluster markers for each AM subpopulation.

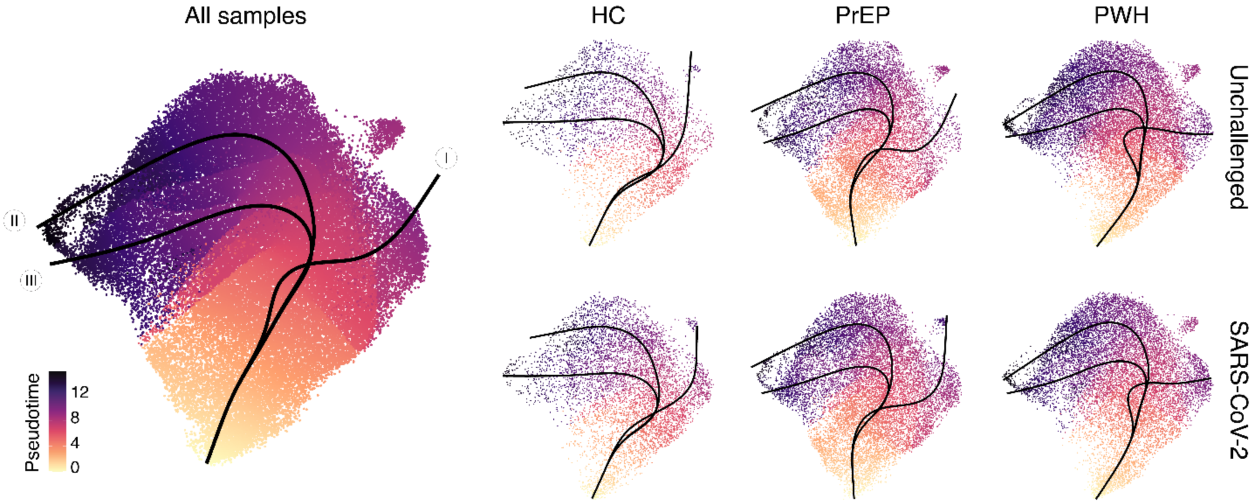

Fig S9. Pseudotime and trajectory analysis.

The starting point of the pseudotime scale and the initial trajectory were obtained unsupervised from the data. Trajectory branches are shown as black lines on top of the UMAP. The left panel shows the trajectory obtained for all cells. The panel on the right shows the trajectory for each group separated by SARS-CoV-2 challenge status.

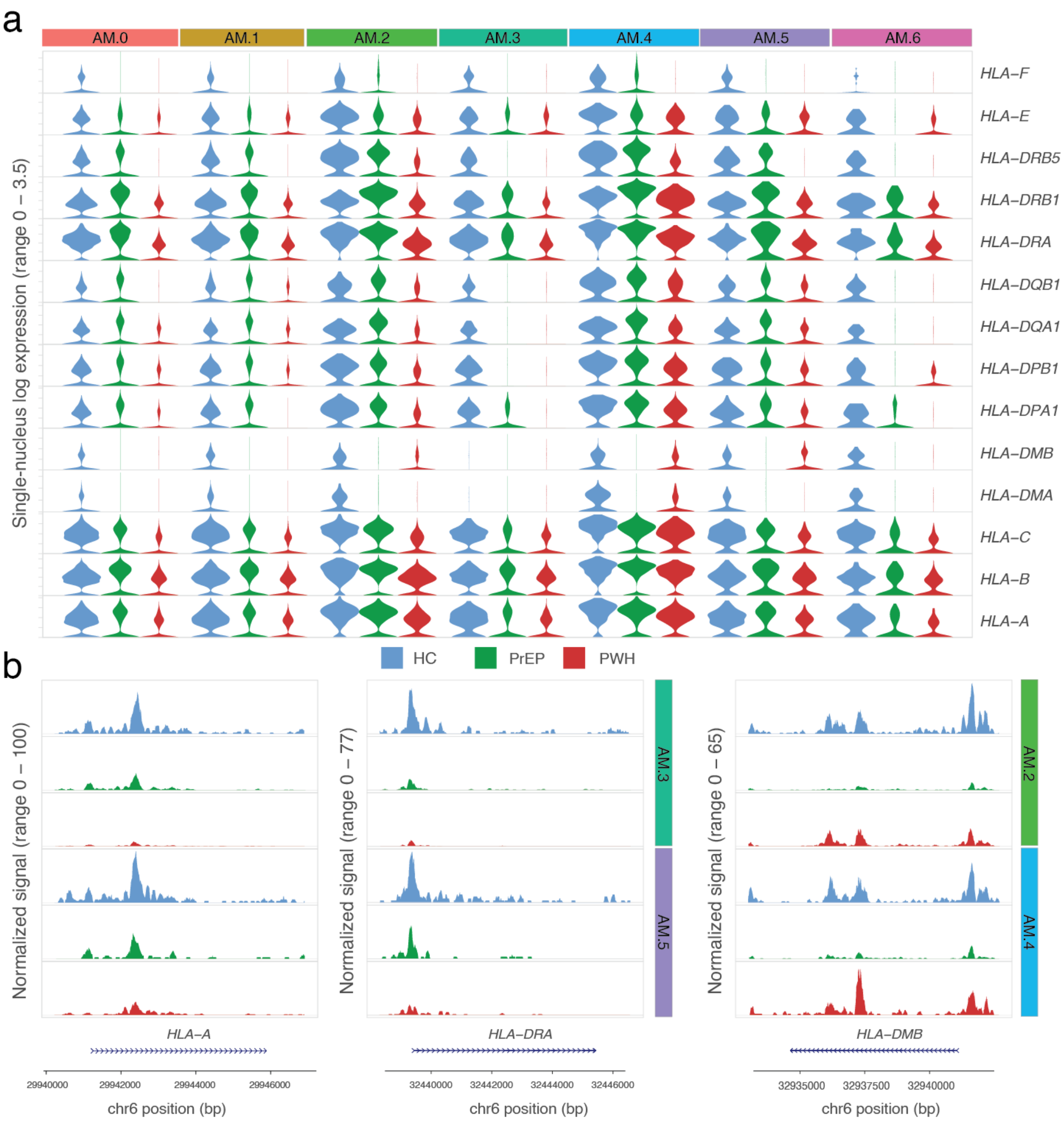

Fig. S10. Constitutive HLA gene expression and chromatin accessibility is impaired in AM from participants on ART.

(a) Single-nucleus expression of HLA genes. Violin plots indicate the per cell expression of the genes listed on the right. (b) Chromatin accessibility plots for the classical class I *HLA-A* and class II *HLA-DRB1* genes in the AM.3 and AM.5 subpopulations and the non-classical class II *HLA-DMB* for AM.2 and AM.4 subpopulations. Tracks display peaks representing baseline chromatin accessibility down sampled to show the same number of cells per group.

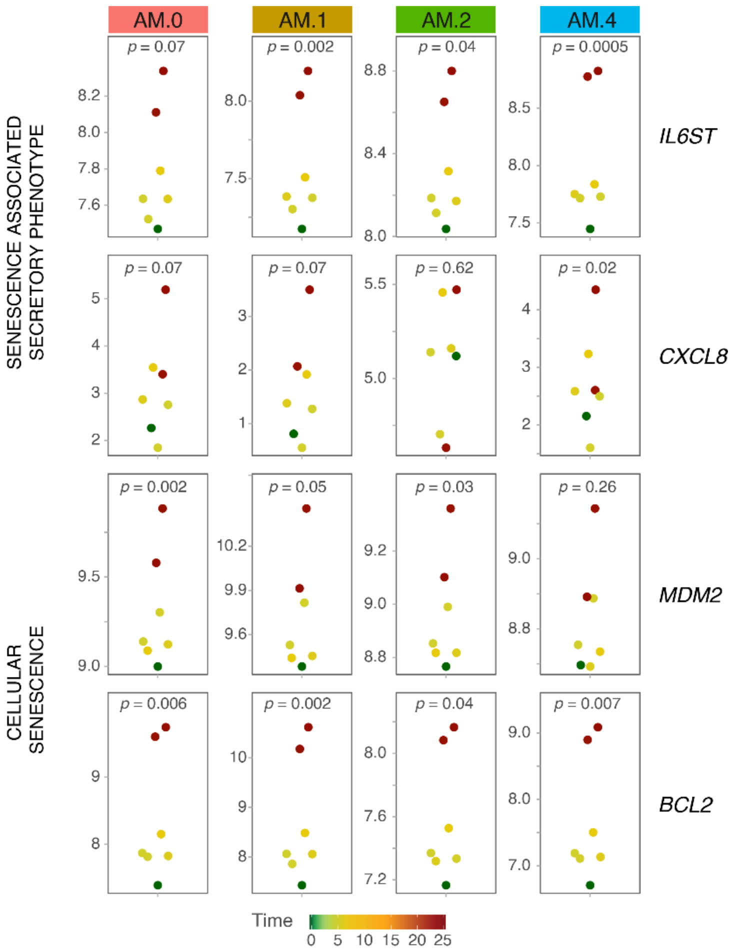

Fig. S11. Senescence markers associate with continuous exposure to ART at the single-nucleus level. Normalized pseudobulk gene expression across timepoints of continuous exposure to ART per AM subpopulation for selected genes from Senescence Associated Secretory Phenotype (SASP) and cellular senescence HALLMARK gene sets.

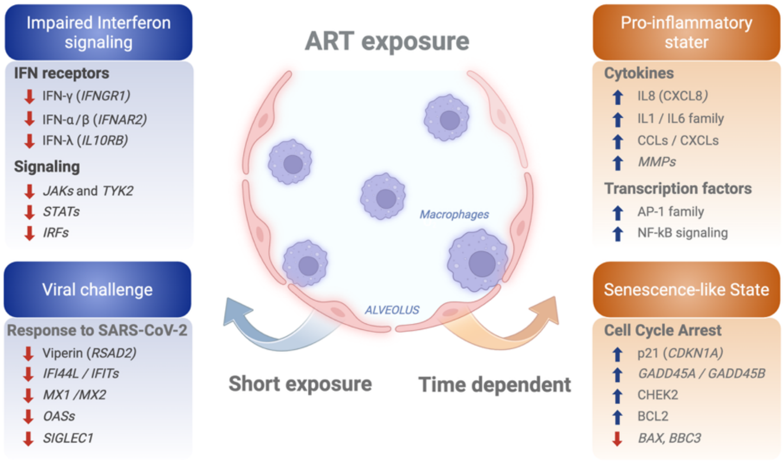

Fig S12. Schematic overview of ART exposure in alveolar macrophages

Short-term exposure to ART in alveolar macrophages is associated with reduced interferon signaling and blunted antiviral responses to SARS-CoV-2 challenge. Long-term exposure to ART induces a pro-inflammatory senescence-like states, with increased activity of inflammation inducing transcription factors, cell-cycle arrest, and signatures secretion of senescence-associated cytokines.
