# Supplementary material for: Continuous antiretroviral therapy induces progressive senescence-like reprogramming of alveolar macrophages
Source: Front Immunol. 2026 Apr 22;17:1805936. doi: 10.3389/fimmu.2026.1805936 (PMC13144088; doi:10.3389/fimmu.2026.1805936)
Supplement: Supplementary file 2 [file DataSheet2.pdf]

Supplementary Methods

Library preparation

Data for bulk RNAseq, ATACseq, and H3K27ac was generated previously and the methodology used to prepare libraries and to process the data for RNA-, ATAC- and H3K27ac was described in (1). For this study, we added two histone marks: H3K4me3 and H3K27me3. We also expanded the dataset for the H3K27ac mark. Libraries for the three histone marks were produced using ChIPmentation kits for histones from Diagenode (C010110009). Aliquots of crosslinked AM containing 10^5 cells stored at -80°C were thawed and resuspended on 50 µL of ice-cold Sarkosyl buffer. Next, samples were sonicated on Bioruptor Pico to obtain fragments between 200 – 500 bp. ChIPmentation was carried out using the automated IP-Star Compact with Diagenode recommended protocol and the Diagenode antibodies for H3K4me3 (C15410003), H3K27ac (C15410196), and H3K27me3 (C15410195). After overnight incubation, DNA fragments captured by the immunoprecipitation were reverse crosslinked and unique barcodes, Diagenode (C01011034, C01011036, and C01011037), were added to the sequences of each subject and histone mark. Finally, libraries were sequenced on Illumina NovaSeq 6000 S4 PE100 aiming for an average of 50 million reads per library. The raw sequences were then processed using the same protocol previously reported (1).

For the snMulti, nuclei preparation was done following an adapted 10xGenomics protocol (CG000365). Specifically, to obtain good quality AM nuclei from BAL cells were lysed while beating with 3mm glass beads for 4 second on MP Biomedicals™ FastPrep-24™ (speed 6.5). Next, ATAC and gene expression libraries were constructed following manufacturer's instructions using 10xGenomics Epi multiome ATAC + Gene Expression kit (CG000338). snMulti libraries were paired-end sequenced on Illumina NovaSeq 6000 S4 flow cells.

Bulk peak to gene assignment

Genes were assigned to peaks using *annotatePeak* from ChIPSeeker v.1.40 (2). Each peak from the epigenetic assays was assigned to the nearest gene and to any additional flanking gene at < 5 kilobases from a peak.

snMulti quality control and data integration

Single-nucleus multimodal sequencing data was processed using 10X cellranger-arc v.2.0.2 with default parameters. Barcoded reads from each single nucleus were aligned to the human genome GRCh38. Data processing was performed per modality prior to multimodal integration. First, HDF5 files with gene expression count were imported into R using BPCells v.0.3.0 (3) and processed with Seurat v5.2.1 (4). Nucleus with mitochondrial reads > 0.2 and detectable genes < 300 were excluded. Next, chromatin accessibility counts and fragments for nucleus that passed quality-control for the RNA modality were imported to Seurat and processed using Signac v.1.14.0 (5). We used a unified peak set across all samples to recalculate feature matrix counts before snATAC integration. We then estimated snATAC parameters and excluded nucleus with fraction of reads in peaks < 0.6, nucleosome signal < 1.5, TSS enrichment < 1, and counts < 1000.

The snRNA modality was integrated using the standard Seurat multiomics pipeline. The data for each library was normalized with SCTransform, and integration was performed using *IntegrateLayers* with reciprocal PCA (RPCA) using one unchallenged library from each group (HC, PrEP, and PWH) as a reference and the top 30 PCs. Dimensionality reduction and clustering were performed using *FindNeighbors, FindClusters*, and *RunUMAP*, to define cells subpopulations and to separate AM from DCs and lymphocytes, which represented between 0.1% and 10% of total cells from BAL. snATAC integration followed Signac pipeline. Term frequency–inverse document frequency (TF-IDF) normalization was applied, followed by latent semantic indexing (LSI) via

RunSVD. The LSI 2 to 15 were used for dimension reduction and clustering. The first LSI component was excluded from the integration as it captures sequencing depth. Cell subpopulations were identified using the same approach as for the snRNA modality. Finally, we performed weighted nearest neighbor (WNN) analysis using Seurat *FindMultiModalNeighbors* to integrate the RPCA and LSI reductions from snRNA and snATAC, respectively. snMulti WNN clusters were used to define the AM subpopulations shown throughout the manuscript.

Single nucleus annotation

To identify the main biological processes characterizing the physiological state of AM subpopulations we used Seurat *FindConservedMarkers* for unchallenged, and SARS-CoV-2 challenged libraries separately. Next, we used cluster markers with positive fold changes expressed in > 25% of the unchallenged AM for each subpopulation to perform a gene ontology (GO) analysis using *enrichGO* form clusterProfiler (6). We selected the most significant GO term per subpopulation as a proxy of the main physiological state of the AM clusters. We then calculated a module score using *AddModuleScore* using the cluster marker genes per nucleus for the top GO terms and indicated the density of the biological state in the UMAP using *plot_density* from Nebulosa v.1.0.1 (7). To identify a transitional state of the AM subpopulations we performed pseudotime and trajectory analysis using unchallenged libraries with slingshot v.2.14.0 (8).

Statistical modeling

Bulk differences in epigenetics and transcriptomics of AM were tested with three models comparing: (i) groups: PrEP, PWH and HC; (ii) time on ART for PrEP and for PWH, and (iii) continuous exposure to ART adjusted by group with the following linear modeling in Limma v3.60.2 (9):

(i) $$Y_{(i,j)} \sim \beta 0_{(i)} + \beta_{Groups} \cdot X_{Groups\ (PrEP,PWH)} + \beta_{Confounders} \cdot X_{(Confounders)} + \beta_{Batches} \cdot X_{(Batches)} + \varepsilon_{(i,j)}$$

(ii) $$Y_{(i,j)} \sim \beta 0_{(i)} + \beta_{Time \times Groups} \cdot (X_{Groups\ (PrEP,PWH)} \times X_{\mathrm{Time}}) + \beta_{Groups} \cdot X_{Groups\ (HC,PrEP,PWH)}$$

$$+ \beta_{Confounders} \cdot X_{(Confounders)} + \beta_{Batches} \cdot X_{(Batches)} + \varepsilon_{(i,j)}$$

(iii) $$Y_{(i,j)} \sim \beta 0_{(i)} + \beta_{Time} \cdot X_{\text{Time}} + \beta_{Group} \cdot X_{Groups\ (HC,PrEP,PWH)} + \beta_{Confounders} \cdot X_{(Confounders)} + \beta_{Batches} \cdot X_{(Batches)} + \varepsilon_{(i,j)}$$

Where $Y_{(i,j)}$ represents the estimate quantification for each feature "i" and sample "j". Briefly, quantification matrices with $Y_{(i,j)}$ were depth-normalized with *calcNormFactors* upperquartile (0.75) for the epigenetic assays and TMM for the transcriptomic data using edgeR v.3.40.2. The mean-variance relationship and log2 count per million was estimated with *voom* v3.60.2 (10). In these models, $\beta 0_{(i)}$ represents the intercept for the feature "i". In model (i) the intercept corresponds to the HC group. In models (ii) and (iii) the intercept represents the predicted value of *Y* when the continuous variables of interest (e.g. Time on ART) are equal to zero. In model (i) β_{Groups} output two β that captures the main effect for PrEP and PWH relative to the intercept (HC), respectively. The contrast between PrEP and PWH was done post-hoc by contrasting their respective β coefficients. In models (ii) and (iii) the β_{Groups} adjusts for the main effect of group membership on the "Time" effect. In model (ii) the $\beta_{Time \times Groups}$ is an interaction term that output two β that captures the effect of time on ART for PrEP and for PWH. In model (iii) the β_{Time} term estimates the effect of continuous exposure to ART adjusting for the main group effect. The coefficients of $\beta_{Confounders}$ included the phenotypic confounders: sex, chronological age, smoking status, *Mtb* challenge, and a seasonal effect of BAL collection. The coefficients of $\beta_{Batches}$ included the technical covariates: sequencing batch, fractions of reads in peaks for the epigenetic assays, time of AM incubation, and number of AM seeded per plate. The $\varepsilon_{(i,j)}$ represents the residual error not explained by variables in the model. A Benjamini–Hochberg (BH) procedure was used to estimate False Discovery Rate (FDR) for each assay in the three tested models. Genes and peaks presenting FDR < 10% were selected for further follow-up on the pathway and transcription factors analyses.

snMulti differential analysis was performed using a pseudo bulk approach per AM subpopulations. Briefly, pseudo counts per AM subpopulations, subject, and challenge status were

calculated using Seurat *AggregateExpression*. Next, genes expressed in less than 20% of the nuclei of a subpopulation for the snRNA modality and peaks quantified in less than 5% of the snATAC modality were excluded from the analysis. For the snATAC pseudo bulk we filtered for peaks linked to genes. Chromatin region from snATAC were assigned to genes using Signac *LinkPeaks*. Peaks that correlated with flanking gene expression within 250kb with a Pearson correlation > 0.01 and *p* value < 0.1 were selected for downstream analysis. We used this lenient cut off to avoid removing epigenetic features that lacked stronger correlations with genes due to the sparsity of the snATAC data. The pseudo counts per modality was performed as in bulk (10, 11). The statistical analyses testing differences between groups and continuous exposure to ART applied the same statistical framework of models (i) and (iii). To test AM response to SARS-CoV-2 challenge per group we used a per subject paired design as indicated in the equation below.

$$\text{(iv)} \quad Y_{(i,j)} \sim \beta 0_{(i)} + \beta_{(\text{Groups (HC, PrEP, PWH)} \times \text{Challenge})} \cdot X_{(\text{Groups (HC, PrEP, PWH)}} \times X_{\text{Challenge})} + \beta_{\text{Time} \times \text{Challenge}} \cdot (X_{\text{Time}} \times X_{\text{Challenge}}) + \beta_{(\text{Subjects})} \cdot X_{(\text{Subjects})} + \varepsilon_{(i,j)}$$

In this model, $\beta 0_{(i)}$ represents the intercept which corresponds to one unchallenged HC library followed by two interaction terms. The first interaction term captures the effect of SARS-CoV-2 challenge with one output β per Group (HC, PrEP, and PWH) and the second interaction term β captures the effect of SARS-CoV-2 according to the time of continuous exposure to ART. Then one β per subject characterizes the blocking design and the $\varepsilon_{(i,j)}$ represents the residual error not explained by variables in the model.

Motif enrichment analysis

To test for enrichment of transcription factor binding motifs in differential regions associated with the continuous exposure to ART, we used *findMotifsGenome* from HOMER v4.11 (12). Differential accessible chromatin regions (ATAC) were compared to (i) 500,000 random regions

matched by size and GC content and (ii) nonsignificant 20,060 regions at FDR > 50% in the continuous exposure to ART analysis. We considered a motif to be significantly enriched if it presented FDR < 1%. Pairwise correlation of TF motif binding by similarities was performed with STAMP v.1.3 default parameters (13).

Footprint analysis

Transcription factor Occupancy also known as Footprint analysis was performed with TOBIAS v0.14.0 using covariate-regressed ATAC traces (14). Briefly, we adapted the concept of regressing out known confounders (e.g. covariables and batch effects) prior to chromatin modeling as proposed in (15, 16). First, signal intensity for regions with increased chromatin accessibility (ATAC) with continuous exposure to ART was calculated from BAM files of each subject using *bamCoverage* from deepTools v.3.5.1 with --binSize 1 and --normalizeUsing CPM (17). Next, ATAC signals per sample were summarized using *multiBigwigSummary* (17). The output of this procedure was a quantification matrix with ATAC signal intensity at 1bp resolution per subject. This matrix was then exported into R v4.4.0. We used *removeBatchEffect* from *Limma* to calculate the residual signal intensity after regressing the coefficients $\beta_{\text{Group (HC,PrEP,PWH)}}$, $\beta_{\text{Confounders}}$ and β_{Batch} while preserving β_0 and $\beta_{Time.}$ from the model (iii). This residual signal intensity matrix represents ATAC traces mimicking the linear model (iii) tested in the differential analysis. The residual traces were exported from R and converted back to individual BigWigs using *bedGraphToBigWig* from UCSC. Next, we used TOBIAS *ScoreBigwig* to identify regions of protein binding across differential regions with continuous exposure to ART. To predict which TF bind to the footprints detected, we combined footprint scores per subject with information on TF binding motifs using TOBIAS *BINDetect*. We used as reference 919 motifs from the JASPAR CORE database for vertebrates (18). *BINDetect* calculates a mean footprint score for each of the 919 TF binding sites split per subject. This score can be compared between samples to access differential changes as TF having high-scoring

footprints denotes a clearer and more defined bound. To test if the TF footprint intensity was associated with time, we fit a linear model comparing the footprint mean intensity per TF with the continuous exposure to ART. Fold change per year was calculated via the exponential of the model slopes and FDR *p*-values were adjusted with BH. TOBIAS *PlotAggregate* was used to summarize traces for selected examples of TF. The residual signal intensities for selected examples of footprints were plotted per timepoint and according to their chromosomal position for each assay using *ggplot2* v.3.4.4 with *geom_spline* from *ggformula* v.0.10.4 (19, 20).

snMulti transcription factor signature and gene regulatory network

We infer TF activity signature using decoupleR v2.4.1 as previously described (21, 22). Briefly, TF – target gene interaction network was obtained from the collecTRI database. We filtered the snRNA modality for genes significantly associated with continuous exposure to ART (FDR < 10%) within the AM subpopulation. TF activity scores were computed per cell using *run_ulm*, which fits a linear model that predicts the observed gene expression based solely on the TF-Gene interaction weights, with the t-value of the slope used as the activity score. To evaluate the relationship between TF activity and continuous exposure to ART, we modeled TF scores as a function of time, adjusting for group (HC, PrEP, PWH) using the *lm* function in base R. β coefficients representing the effect of continuous exposure to ART on TF activity were extracted, and a BH FDR was calculated. TFs showing absolute $\beta > 0.02$ and FDR < 0.01 were considered significant.

To infer the multimodal TF gene regulatory networks (GRN) associated with continuous exposure to ART, we applied the Pando v1.0.2 framework (23). Pando models gene expression as a function of the interaction between TF expression and chromatin accessibility at regions with TF binding sites per nucleus. For the GRN analysis, we tested 627 TFs from the JASPAR2020 human CORE collection (24). As candidates for the GRN, we evaluated all differential features for snRNA (n

= 867) and snATAC (n = 3,332) associated with continuous exposure to ART. Regulatory TF interactions were modeled with *infer_grn* using the Signac peak-to-gene method, a 250 kb upstream and 50 kb downstream window, and multiple testing correction using the BH procedure. Modules of co-regulated genes were identified using *find_modules*, and modules with FDR < 10% were considered significant.

REFERENCES

1. Correa-Macedo W, Fava VM, Orlova M, Cassart P, Olivenstein R, Sanz J, et al. Alveolar macrophages from persons living with HIV show impaired epigenetic response to Mycobacterium tuberculosis. J Clin Invest. 2021;131(22).
2. Yu G, Wang LG, He QY. ChIPseeker: an R/Bioconductor package for ChIP peak annotation, comparison and visualization. Bioinformatics. 2015;31(14):2382-3.
3. Parks B, Greenleaf W. Scalable high-performance single cell data analysis with BPCells. bioRxiv. 2025:2025.03.27.645853.
4. Hao Y, Stuart T, Kowalski MH, Choudhary S, Hoffman P, Hartman A, et al. Dictionary learning for integrative, multimodal and scalable single-cell analysis. Nature biotechnology. 2024;42(2):293-304.
5. Stuart T, Srivastava A, Madad S, Lareau CA, Satija R. Single-cell chromatin state analysis with Signac. Nature methods. 2021;18(11):1333-41.
6. Yu G, Wang LG, Han Y, He QY. clusterProfiler: an R package for comparing biological themes among gene clusters. OMICS. 2012;16(5):284-7.
7. Alquicira-Hernandez J, Powell JE. Nebulosa recovers single-cell gene expression signals by kernel density estimation. Bioinformatics. 2021;37(16):2485-7.
8. Street K, Risso D, Fletcher RB, Das D, Ngai J, Yosef N, et al. Slingshot: cell lineage and pseudotime inference for single-cell transcriptomics. BMC genomics. 2018;19(1):477.
9. Ritchie ME, Phipson B, Wu D, Hu Y, Law CW, Shi W, et al. limma powers differential expression analyses for RNA-sequencing and microarray studies. Nucleic Acids Res. 2015;43(7):e47.
10. Law CW, Chen Y, Shi W, Smyth GK. voom: Precision weights unlock linear model analysis tools for RNA-seq read counts. Genome Biol. 2014;15(2):R29.
11. Robinson MD, McCarthy DJ, Smyth GK. edgeR: a Bioconductor package for differential expression analysis of digital gene expression data. Bioinformatics. 2010;26(1):139-40.
12. Heinz S, Benner C, Spann N, Bertolino E, Lin YC, Laslo P, et al. Simple combinations of lineage-determining transcription factors prime cis-regulatory elements required for macrophage and B cell identities. Molecular cell. 2010;38(4):576-89.

13. Mahony S, Auron PE, Benos PV. DNA familial binding profiles made easy: comparison of various motif alignment and clustering strategies. PLoS Comput Biol. 2007;3(3):e61.
14. Bentsen M, Goymann P, Schultheis H, Klee K, Petrova A, Wiegandt R, et al. ATAC-seq footprinting unravels kinetics of transcription factor binding during zygotic genome activation. Nature communications. 2020;11(1):4267.
15. Zou J, Ernst J. Chromatin state modeling across individuals reveals global patterns of histone modifications. bioRxiv. 2022:2022.08.02.502571.
16. Grubert F, Zaugg JB, Kasowski M, Ursu O, Spacek DV, Martin AR, et al. Genetic Control of Chromatin States in Humans Involves Local and Distal Chromosomal Interactions. Cell. 2015;162(5):1051-65.
17. Ramirez F, Ryan DP, Gruning B, Bhardwaj V, Kilpert F, Richter AS, et al. deepTools2: a next generation web server for deep-sequencing data analysis. Nucleic Acids Res. 2016;44(W1):W160-5.
18. Rauluseviciute I, Riudavets-Puig R, Blanc-Mathieu R, Castro-Mondragon JA, Ferenc K, Kumar V, et al. JASPAR 2024: 20th anniversary of the open-access database of transcription factor binding profiles. Nucleic Acids Res. 2024;52(D1):D174-D82.
19. Wickham H. ggplot2: Elegant Graphics for Data Analysis: Springer Publishing Company, Incorporated; 2009. 216 p.
20. Kaplan D, Pruim R. ggformula: Formula Interface to the Grammar of Graphics. https://githubcom/ProjectMOSAIC/ggformula. 2023.
21. Badia IMP, Velez Santiago J, Braunger J, Geiss C, Dimitrov D, Muller-Dott S, et al. decoupleR: ensemble of computational methods to infer biological activities from omics data. Bioinform Adv. 2022;2(1):vbac016.
22. Dallmann-Sauer M, Fava VM, Malherbe ST, MacDonald CE, Orlova M, Kroon EE, et al. Mycobacterium tuberculosis resisters despite HIV exhibit activated T cells and macrophages in their pulmonary alveoli. J Clin Invest. 2025;135(7).
23. Fleck JS, Jansen SMJ, Wollny D, Zenk F, Seimiya M, Jain A, et al. Inferring and perturbing cell fate regulomes in human brain organoids. Nature. 2023;621(7978):365-72.
24. Fornes O, Castro-Mondragon JA, Khan A, van der Lee R, Zhang X, Richmond PA, et al. JASPAR 2020: update of the open-access database of transcription factor binding profiles. Nucleic Acids Res. 2020;48(D1):D87-D92.
